# Gut physiology meets microbiome science

**DOI:** 10.1017/gmb.2022.10

**Published:** 2022-12-12

**Authors:** Hannelore Daniel

**Affiliations:** ex. School of Life Sciences, Technical University of Munich, Gregor-Mendel-Strasse 2, 85354 Freising, Germany

**Keywords:** gastrointestinal tract, microbiome, physiology, rodents, diet, in situ

## Abstract

Research on the gut microbiome has gained high popularity and almost every disease has meanwhile been linked to alterations in microbiome composition. Typically assessed via stool samples, the microbiome displays a huge diversity with a multitude of environmental parameters already identified as contributing to its character. Despite impressive scientific progress, normal microbiome diversity remains largely unexplained and it is tempting to speculate some of the yet unexplained variance is hidden in normal gut physiology. Although a few genome/phenome-wide associations studies have recently highlighted physiological parameters such as stool frequency, known as contributing to microbiome diversity, there is a large knowledge base from decades of basic research on gut functions that can be explored for possible links to stool features and microbiome characteristics. And, when extrapolating findings from faecal samples to the biology in the intestinal lumen or the mucosal microenvironment, gut anatomy and physiology features need to be considered. Similarly, differences in anatomy and physiology between rodents and humans need attention when discussing findings in animals in relation to human physiology and nutrition.

## Introduction

The microbiome emerged as new term and research area about two decades ago and has since proliferated in an unseen manner. However, research on bacteria residing in the mammalian gastrointestinal tract goes back more than a century with important findings generated on bacterial density, diversity, and metabolic capacities. Many of these findings appeared almost forgotten and for decades the “gut flora” was not perceived as of major relevance for human health or disease. Yet, in animal science gut bacteria became an area of high interest even before microbiota research became popular in the biomedical field because of the wide use of antibiotics in farm animals and the need to substitute these. Recent years have produced an enormous number of high-ranking publications around the gut microbiome, and it is almost impossible to comprehend the published material. What frequently comes short in microbiome science is some fundamental gut physiology. It is the ambition of this review to address issues around normal gastrointestinal functions that directly or indirectly affect the quantity and composition of the gut microbiome as determined usually in stool samples.

## The gut is a masterpiece of biological complexity

**Figure 1:**
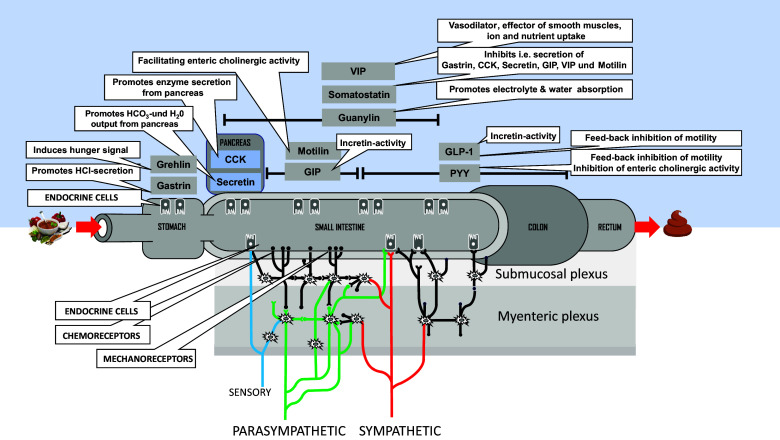
Gastrointestinal tract and it’s neuro-endocrine control system. A multitude of sensor cells with different densities in longitudinal direction coupled to the enteric nervous system (lower part) and the endocrine system with a large repertoire of hormones/mediators (upper part) mediate tight control of every step in intake and processing of food as well as absorption of the constituents embedded into bidirectional communication with peripheral organs and brain.

The gastrointestinal system (GIS) is the interface between the food environment and the host metabolic system. Because of the uncertainty of whether food is or becomes available, the GIS requires a repertoire of sensors to register the incoming food and its composition and to synchronise digestion and absorption of food constituents with the need of metabolism for optimal homeostasis. Sensory inputs are realised by a variety of receptors (mainly G-protein-coupled receptors) and transporters expressed on the surface of specialised enteroendocrine cells (EECS) found in different densities throughout the GIS. Perception of individual nutrients (sugars, fatty acids, amino acids and peptides) in the gut is translated into hormone secretion from EECS or into activation of enteric sensory neurons with signals for the gut but also various organs including brain (Figure 1).

EECS and some other cells in the gut produce a large panel of peptide hormones and mediators derived from amino acids and lipids which in essence control every step in the processing of food. Gastric emptying and intestinal transit are key in adjusting the substrate load to the capacity for digestion and absorption. This includes the secretion of HCl and some enzymes in stomach mediated by gastrin as well as HCO_3_^−^ secretion from exocrine pancreas elicited by secretin. Enzyme output from pancreas as well as contraction of the gallbladder with the release of bile acids into the lumen are initiated by CCK. Ghrelin secretion from stomach is simultaneously inhibited by the incoming food and together with secretin and CCK those three hormones are important for short-time satiety effects in brain. In addition to GLP-1 secreted from L-cells, gastric insulinotropic peptide (GIP) secreted from K-cells affects the postprandial glucose profiles in systemic circulation as well as insulin output from the endocrine pancreas (Gasbjerg et al., 2019; Mayendraraj et al., 2022).

Food composition and its quantity affect the gastrointestinal response to a meal with major changes in the motility as this adjusts the residence time of the food bolus in stomach and intestine to limit the substrate load for the digestive and absorptive processes. Energy density of the ingested food is a critical determinant for the rate of gastric emptying that in turn affects the extend of digestion and velocity of absorption in duodenum and jejunum. This becomes visible by the addition of fat to an oral glucose bolus which can almost completely abolish the glycaemic response when compared to intake of sugars/carbohydrates without any other high-energy constituent (Gentilcore et al., 2006). This “caloric load effect” sustains over the entire small intestine and mediates also the so-called ileal break. When higher amounts of nutrient energy reach the terminal ileum, the release of PYY and GLP-1 is increased (Spiller et al., 1988; van Avesaat et al., 2015; Vu, 2007) and these two hormones cause a slowing down of gastric emptying and intestinal transit time, possibly via an inhibition of release of acetylcholine and/or via an opioid-pathway (Zhao et al., 2000). This can be interpreted as a measure to optimise energy extraction in the small intestine and to prevent a “loss” of energy to colon.

Transit time through the small intestine is also a major factor in maintaining a low bacterial density in this segment. A rather fast transit of the chyme accounting to 2 to 8 h for mouth to cecum (mean value with variation based on sex, age and diet) contributes to lower bacterial counts in human jejunum and ileum (Arhan et al., 1981; Probert et al., 1995). By the detergent quality and the high luminal concentration of bile acids (BA) in the upper small intestine (of 20–30 mM) after food intake (Sonne et al., 2014), BA also contribute to control of bacterial density. Gastric acid secretion is also considered to play a role as revealed by the wide use of proton-pump-inhibitors that were shown to promote *Clostridium difficile* infections and change the intestinal microbiome composition (Naito et al., 2018). Based on the principle that energy extraction in the small intestine should be optimal with motility adapted accordingly, the question arises on the quantity and quality of the “feed” of the microbiota in the large intestine. Only very limited information is available from ileostomy patients and analysis of the emptied contents for an estimation of the quantity and quality of substrates that become available for bacterial growth in the large intestine. Although animal studies would allow to collect intestinal contents at given time points from the different regions of the small and large intestine, we need to be careful when translating the findings from rodents into the human situation.

## The mouse is unfortunately not a tiny human and mice feed is not human food

At that point a short excursion into gut anatomy and physiology of mouse and human intestine needs to be made. There are major differences in the overall structure and morphology as well as the organisation and length of the various parts of the GIS of the mouse small and large intestines that require consideration when findings in mice are transferred to the human condition. Although the relative surface area of the intestine to total body surface area is similar between two species, the small intestine to large intestine length ratio is approximately 2.5 to 1 in mice and approximately 7 to 1 in humans (Nguyen et al., 2015). That sheds light on the smaller contribution that the small intestine appears to have in overall energy and nutrient extraction from food in mice as compared to humans. In addition, the feed provided to mice is either irradiated or autoclaved in dry state, with the majority of starch either still crystalline or partly retrograded. The type of starch and its gelatinisation state are affecting digestibility (Böswald et al., 2021; Holm et al., 1988). The processing of laboratory diets thus determines to which extent fermentable substrates reach the microbiota in ileum and colon. When animals are sacrificed shortly after food intake, large junks of the pellet food can be recovered from cecum, demonstrating the limited processing of the pellet junks in the small intestine. The mouse cecum has the character of a large fermentation chamber but that corresponds in humans only to the lower part of the ascending colon. What in addition makes the mouse quite different is the very fast intestinal transit. By use of a ^99m^Technetium labelled marker bolus, it was demonstrated that already after 1 h the tracer had entered the cecum and colon and after 6–7 h the majority of the tracer had left the large intestine (Padmanabhan et al., 2013). That contrasts with the transit in the human GIS with 3–6 h for the small intestine and a mouth-to-anus time of 48–120 h (Maharaj and Edginton, 2016; Probert et al., 1995). Taken together, there are significant differences in the time and location of digestion of food and in absorption of nutrients and energy between mice and humans. Whereas humans digest and absorb mainly in the small intestine, in mice transit is too fast and the food much harder to digest by pancreatic enzymes with a larger proportion of energy and nutrients delivered across the ileocecal valve and extracted from cecum/colon after fermentation. Changes in the cecal/colonic microbiome caused by diet may thus have more impact on the animal’s metabolism and health status. That applies as well to germfree/gnotobiotic animals or animals receiving antibiotics in which energy extraction may be impaired by loss of bacteria. What should also not be forgotten, is that rodents perform coprophagy which makes certain nutrients (ie, vitamins) better available by providing those to the upper small intestine. That the metabolism of bacteria in cecum contributes with heat production to the animal’s body temperature has recently been revealed (Riedl et al., 2021) with an estimated 8 per cent of total energy expenditure. Although the vital bacterial biomass in human colon is not known, in mice, cecum content accounts for approximately 1 per cent of total body mass (Drew et al., 2018) whereas in humans, colonic contents of approximately 230 g wet weight represent less than 0.35 per cent of body mass (Cummings et al., 1990). That may mean that heat generated in human colon is of only minor relevance for energy balance – but even small differences may in long-term have consequences for body and fat mass.

## Microbiome needs and return

Bacterial reproduction and growth require energy. The bacterial biomass in human large intestine is much smaller than originally claimed (Cummings et al., 1990; Sender et al., 2016) and may range between 100 and 200 g material (wet weight) in an individual living in an industrialised country. Total stool volume produced per day varies from high to low-income countries from 126 to 250 g wet weight and 28 to 38 g dry weight (Rose et al., 2015). Any comparison, eg, of bacterial diversity across developed and rural countries and populations should take that into account. Approximately 15 g bacterial mass is excreted with the faeces per day in developed countries (Stephen and Cummings, 1980) which needs to be replaced in a steady state. Ignoring possible differences in energy demands between aerobic and anaerobic bacteria, approximately 0.5 MJ would be required (rough estimate) per day for substitution of 15 g bacterial biomass (Chen, 1964). Besides some secretion of solutes into colon from endogenous sources, the majority of bacterial substrates enter from the ileum. In healthy ileostomy patients on a standard diet containing approximately 20 g of fibre, stoma output revealed that approximately 1.3 MJ per day leave ileum and that quantity increased on a high fibre bran-based diet to 2.4 MJ (Isaksson et al., 2013). The amount of nutrients and energy delivered to colon thus varies considerably based on diet constituents and the processing state of the food.

This energy passing from the small intestine into colon comprises small quantities of carbohydrates, lipids and proteins as well as fibres from the food consumed, but there is also a constant flow of mucus and other glycoproteins from all secretions into the GIS. In addition, larger quantities of urea but also glucose and some minor quantities of other solutes are constantly reaching the GIS. The latter are all substrates for maintaining the microbiome even when the host is fasting or starving. That this endogenous provision of “feed” for the bacteria is of relevance have two recently published cohort studies with thousands of participants convincingly demonstrated. Both studies revealed a marked influence of the blood group antigens (AB0 system) and secretor status on microbiome diversity (Esteban et al., 2022; Qin et al., 2022). All cell surface proteins but also mucus released from goblet cells and contained in secretions such as saliva carry distinct glycan structures with signatures like the blood group antigens. These glycans resist hydrolysis in the small intestine because of lack of specific glucosidases leading to a constant provision of a characteristic pattern of oligosaccharides to the colon. These glycans comprising sugars such as galactose, fucose, *N*-acetyl-glucosamine and *N*-acetyl-galactosamine and others provided to the bacteria lead to significant differences in microbiota-signatures as shown for the blood group glycans. The quantity of sugars bound in the glycans that reach the colon is difficult to assess but may be in the range of 2 to 15 g per day (Johansson et al., 2013; Leal et al., 2017).

Gastrointestinal transit time also plays an important role for the quantity of chyme entering colon (Stephen et al., 1987). Moreover, transit time was also shown to closely associate with the diversity and quantity of bacteria excreted with faeces. With compounds that increase transit time, more fermentable substrates enter the colon, and more bacteria are found in faeces whereas compounds that slow transit have the opposite effects (Stephen et al., 1987). Transit time also affects the water content in colon and in stool and that has been shown to be a relevant determinant of microbiome diversity in humans (Roager et al., 2016; Vandeputte et al., 2015). Since on average approximately 650 kJ of energy are excreted in faeces, only approximately 500 kJ can be predicted as absorbed from colon and that is mainly in form of short-chain organic acids. This rather small quantity of calories becoming available for the host asks for its overall importance in body weight control.

Several studies have in the initial phase of modern microbiome research addressed the contribution of the microbiome in the development of obesity and those were fostered by studies in germfree mice that showed resistance to diet-induced obesity. Those findings were later questioned by studies that failed to demonstrate such a resistance to obesity-development using different “obesogenic diets” in germfree mice (Moretti et al., 2021).

That the microbiome in its diversity is associated with the BMI of individual’s has been observed in large cohorts (Zhernakova et al., 2016). Although human studies also reported significant microbiome differences between lean and obese individuals (Chierico et al., 2018; Le Chateliere et al., 2013), over the years it has become clear that the heterogeneity found in microbiomes of seemingly healthy people is far larger than the characteristics of the microbiomes of obese and lean individuals (Boscaini et al., 2022). As described above, the contribution of the microbiome and its importance for the provision of calories to the host seems small but could in long-term – over decades – make a difference. Yet, the research on diets to alter the microbiome has also not delivered the expected effects. Even well-established fermentable fibres known for improvement of obstipation or intestinal discomfort failed to demonstrate marked changes in the microbiome diversity by when provided over weeks in relevant doses (Canfora et al., 2017; Vandeputte et al., 2017). A consistent finding in these studies however was a substantial increase in the density of *Bifidobacteria*, which also can be increased in density by dietary lactose but here microbiome diversity then depends on the individual capability to digest lactose as large cohorts have recently revealed (Esteban et al., 2022; Qin et al., 2022).

What is striking is that human and animal science appear to look at the contribution of the gut microbiome in body weight gain or body composition with different views. In animal science the wide use of antibiotics was also driven by findings that this increased weight gain – defining antibiotics as growth promotors and that was demonstrated in pigs and chicken (Angelakis, 2017; Gaskins et al., 2002). In pigs the application of different antibiotics led to a reduction in the total quantity of bacteria and to changes in the spectrum of bacteria in ileum as revealed by sequencing (Collier et al., 2003). The amount of energy utilised by bacteria and becoming available for the host as weight gain upon antibiotic treatment was estimated to be approximately 6 per cent of the total food energy. That is in essence identical to the estimated heat production by the caecal microbiome in mice as part of the energy needed to maintain body temperature (Riedl et al., 2021). The increased weight gain found when the microbiota is reduced in density by antibiotics has been attributed to lower energy costs for mucus production and for immune defence in the intestine since fewer bacteria could translate into reduced synthesis of mucus and immune cells and possibly a lower epithelial turn-over. In an impressive paper it was recently demonstrated by use of a stable isotope labelling technique and proteome analysis allowing quantification of protein synthesis rates and protein half-lives that there are major differences between conventional and germfree mice (Arike et al., 2020). Since the intestine is the organ with the highest cell renewal rate it has to have a huge capacity for synthesis of proteins. The proliferation and apoptosis rates underlying cell replacement in the intestine revealed in the labelling experiment a spatial gradient from proximal to distal linked to the different architecture of the epithelia. However, the study also revealed that germfree mice have lower protein synthesis rates including those of the mucus-proteins (Arike et al., 2020). These findings support the notion that bacteria and other organisms and viruses that collectively constitute the ecosystem in the gut, drive cell proliferation, immune defence mechanisms and mucus production rates. The latter seems particularly enforced by bacteria that reside in the mucus layer and that can utilise the glycoproteins as substrates for growth. One very prominent species is *Akkermansia muciniphila* that has frequently been associated with a lean host phenotype and a variety of health benefits (Cani et al., 2022). Stimulation of mucus production in the gut means that the host needs to invest more energy into synthesis of the glycans and proteins and the secretory machinery and that could in life-long perspective affect host body mass.

## Small molecules go across

The production of large quantities of short-chain fatty acids (SCFA) by the microbiota has been demonstrated more than 50 years ago and their role in energy metabolism but also in protecting colonic tissue from malign transformation received a lot of attention; for a recent review see Blaak et al., 2020. Although moderate increases in SCFA levels in faecal samples in human trials using fermentable substrates were demonstrated (Puhlmann et al., 2022), not all intervention studies have consistently observed such changes, even not when high quantities of fibres of up to 45 g per day (Oliver et al., 2021) or 12 g/day of galactooligosaccharides (Canfora et al., 2017) as highly fermentable substrates were consumed by human volunteers. In a study with obese and prediabetic volunteers treated for 7 days with the antibiotics Amoxicillin or Vancomycin only Vancomycin reduced significantly faecal levels of acetate and butyrate but not Amoxicillin. However, antibiotic treatment failed to change fasting plasma SCFA levels (Reijnders et al., 2016) and metabolic phenotyping revealed no clinically relevant effects in the volunteers despite marked changes in the microbiomes.

**Figure 2:**
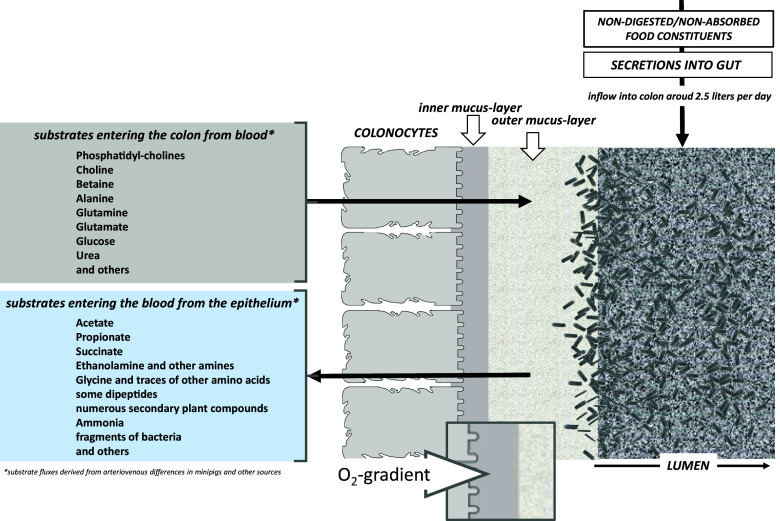
Bidirectional fluxes of substrates/intermediates (compiled from literature) across the colonic tissue from blood to lumen and *vice versa* on background of the epithelial morphology that creates a distinct surface compartment between the cells and the colonic lumen hosting the microbial ecosystem.

Whereas SCFA concentrations in faecal samples reach ~50 mmol/kg (total) dominated by acetate, those in peripheral blood are in the μmolar range represented by acetate (~50 μM), propionate (~5 μM) and butyrate (below 1 μM). SCFA levels in portal blood in humans undergoing elective cholecystectomy for gall stones (Peters et al., 1992) found ~130 μM of acetate, 35 μM of propionate and ~ 17 μM of butyrate. This demonstrates that SCFA analysis in peripheral blood does not reflect production in the gut due to efficient hepatic extraction. Whereas butyrate has manifold effects in colonocytes and also serves as energy substrate in these cells, propionate and acetate are metabolised mainly in liver (den Besten et al., 2013). Whereas propionate can serve as a gluconeogenic substrate, it also can reduce expression of lipogenic enzymes in hepatocytes (Yu et al., 2019) but may itself be incorporated into de novo synthetised fatty acids leading to odd-chain products. Acetate as well serves as substate for de novo synthetised fatty acids or cholesterol and for fatty acid elongation in liver but has also been demonstrated to possess a variety of other biological effects; both, beneficial or harmful (Moffett et al., 2020 **)**. SCFA have also been shown to bind to the G-protein-coupled receptors GPCR41 (FFAR3) and GPCR43 (FFAR2) to alter intracellular signalling – mainly via cAMP (Le Poul et al., 2003, Park et al., 2019). However, the apparent affinities of acetate and propionate for receptor activation are rather low and based on their levels in peripheral or even in portal blood, relevant effects in systemic circulation in humans are likely limited. In addition, acetate and propionate compete with the ketone body acetoacetate in binding to GPCR43 which becomes more important when the mammal is in fasting state and acetoacetate levels raise (Miyamoto et al., 2019; Figure 2).

Although acetate, propionate, butyrate and lactate make up the majority of solutes produced by microbiota with a total concentration of above 120 mmol/L in the gut lumen, many other organic acids and many amines are produced by microbiota as well. A study in obese Yucatan minipigs on a high fat/high sucrose diet with catheters placed in the abdominal artery aorta, the portal vein and the hepatic vein allowed the arteriovenous balance of metabolites across the intestine to be analysed (Poupin et al., 2019). The prominence of acetate and propionate released by the gut was confirmed in this model, but it was also observed that ethanolamine and amino acids such as alanine, phenylalanine or glycine were released from the gut into blood. However, the origin of these amino acids is not clear; it could be the microbiome or just the tissue releasing those amino acids into blood from the overall amino acid turnover in epithelial cells. To answer whether the microbiome brings these substrates into blood, germfree mice seem to be the best model to study. A recent metabolomics study compared serum, brain and faecal samples obtained from germfree and conventional mice with a comprehensive metabolite coverage (Lay et al., 2021). Not unexpected, brain tissue showed only modest changes in a few metabolites and most interestingly there was no change in the classical brain neurotransmitter profile. That means that despite prominent levels of DOPA, serotonin but also GABA and histamine found in faecal samples those appear not to change in concentration in brain when comparing tissues from germfree and conventional mice. This argues against a significant direct communication axis of these transmitters found in colon and faeces and brain. Moreover, the blood–brain barrier has a low intrinsic permeability for neurotransmitters as their transporters are either not expressed here or only efflux-systems from brain are found (Nałęcz, 2017). Bidirectional communication between gut and brain is mainly realised by a multitude of anatomically fixed neuronal connections as well as by numerous hormones produced in the gastrointestinal tract that can enter brain and by hormones produced along the hypothalamic-hypophyseal axis. Vagus nerve endings in the gut may be able to sense the local environment but these afferent projections do not cross the epithelial barrier (Bonaz et al., 2018) and thus require compositional changes in the subepithelial compartment. Those may include alterations in the concentrations of SCFA or bile acids as suggested by the presence of the bile acid receptor TGR5 receptor in myenteric neurons (Alemi et al., 2013).

However, that the brain can sense the presence of bacteria in the intestinal system based on NOD2 in certain brain regions – mediated by muramyl-peptides derived from the peptidoglycans of bacterial cell walls – has recently been demonstrated (Schneider and Thaiss, 2022). Moreover, EECS express toll-like receptors (TLR) for recognition of flagellin, lipopolysaccharide, double-stranded DNA or other bacterial products and even vagal afferents may be able to sense those directly via TLR4 shown to be expressed in the visceral afferents (Raybould, 2010).

Because metabolomics applied to faecal samples revealed also a variety of essential nutrients like amino acids or vitamins, it has been speculated that the microbiome contributes also to the body needs of these indispensable compounds. Of course, bacteria can synthesise essential compounds such as vitamins, but to which extend those contribute to the host vitamin status, is not well defined. Evidence of a contribution to the biotin status comes from the findings that only raw avidin – that binds biotin as the strongest known non-covalent binding – can prevent biotin to be absorbed including that produced by bacteria which then causes clinically relevant biotin-deficiency (Said, 2002). There is also evidence for synthesis of folate and menaquinones (Conly and Stein, 1992; Engevik et al., 2019). Other vitamins are likely also found at low levels when analysing bacteria/stool but here it needs to be defined of whether those vitamins are bioavailable and absorbed in colon. In rodents with extensively exercising coprophagy these vitamins contribute to the vitamin status as they can be absorbed in the small intestine, but in humans, it is extremely difficult to assess the overall contribution of the microbiome to the vitamin status. Although transporters required for absorption of some vitamins have been found in the colonic epithelium, their density seems rather low, and their relevance thus remains elusive.

The limited bioavailability of most solutes in the large intestine provides host protection, eg, for nitrogen or sulphur compounds considered as harmful when entering the systemic circulation in larger quantities (Florin et al., 1991). However, nitrogen but also sulphur availability are most critical for bacterial growth and the maintenance of the biomass in the large intestine. Carbon sources are manifold but synthesis of amino acids and proteins, but also of other biomolecules require larger quantities of nitrogen, and also sulphur. There is a considerable nitrogen flux through the intestinal system with a significant role of the microbiome and that refers particularly to bacteria expressing urease with the ability to hydrolyse urea and the release of bicarbonate and ammonium. This process allows bacteria to build their own microenvironment with high buffering capacity by urea hydrolysis but with need that the host must re-synthetise urea in liver for detoxification of the ammonia with a large demand of ATP. Portal blood ammonia concentrations exceed those in blood that leaves the liver by three to four times – demonstrating the large hepatic capacity for extraction of NH_3_/NH_4_^+^ from the portal system followed by hepatic urea output. Urea recycles into the gut via all fluids secreted into the intestine and via dedicated urea-transporters of the aquaporin-family of membrane channels found throughout the intestinal epithelia. It has been estimated that up to 100 mmol of urea may cycle between host liver and microbiome (Fuller and Reads, 1998).

Whether amino acids produced by the microorganisms in the large intestine can also be taken up by colonic cells into the host is suggested by labelling experiments in piglets that received orally ^15^N-ammonia or labelled urea. The nitrogen is incorporated into amino acids synthetised by the bacteria including indispensable amino acids such as lysine and threonine (Darragh et al., 2017). However, absorption into the body seems rather low (Metges, 2000; van der Wielen et al., 2017). A surprising finding was that the epithelium in mouse and human colon and even rectum expresses the peptide transporter PEPT1 (Wuensch et al., 2013). This carrier protein is found at high levels apical membranes of epithelial cells in the upper small intestine and transports in essence all possible 400 different di- and 8000 possible tripeptides into cells. Although it was demonstrated that it is functional in colon, it is still not known of whether it contributes to amino acid uptake by transport of peptide substrates provided by the microbiota. However, it had also been proposed that PEPT1 can in colon absorb muramyl-peptides as products of bacterial walls and that this would further promote chronic inflammation in intestinal bowel diseases (IBD) such as Crohn’s disease or colitis (Merlin et al., 2001). Yet, when mice lacking the peptide transporter were submitted to different treatments that initiate IBD, no difference to control animals in disease severity could be found (Wuensch et al., 2014) questioning the role of PEPT1 as an enhancer of IBD. There is good evidence that the composition of the microbiome and products thereof play a critical role in IBD; although a taxonomic IBD signature in patients could not yet be identified because of the huge diversity of the human gut microbiome in healthy individuals (Metwaly et al., 2022).

## From faeces back into the gut

When human stool samples are analysed, the microbes found comprise a mixture of dead and living bacteria (with a 1:1 relationship) and the sample reflects mainly the conditions in the descending colon from which the contents are moved into rectum for elimination from the body. In the descending colon the contents are solidified by reabsorption of electrolytes and water. The extent of water left in faeces has been shown to associate with microbiome diversity (Vandeputte et al., 2015). The Bristol stool scale (BSS) classifies the physical appearance of stool by colour and consistency (and thus water content) and ranks always very high amongst the parameters underlying microbiome variance (Falony et al., 2018). Amongst the thousands of publications on human microbiota only a few assessed bacterial communities in different locations of the intestine. Samples collected from the intestine rather than from stool revealed significant differences – even within the human large intestine – in bacterial density and in composition (Martinez-Guryn et al., 2019). Analysis of the colon of deceased organ transplant donors, demonstrated marked differences in microbiome patterns between cecum, colon transversum and sigmoid (James et al., 2020) with an increasing density of Actinobacteria and decreasing density of Proteobacteria towards the sigmoid. But there is not only a longitudinal gradient in density and bacterial spectrum but also a radial segmentation (Donaldson et al., 2016). This may be best explained by physical determinants in which flow through a tubing always shows faster speed in the centre and reduced flow velocity towards the wall. In addition, the mucus layer in its dimensions and its quality contributes to this phenomenon since it represents an “unstirred layer” compartment of several hundreds of microns in thickness. The mucus layer in colon can be as thick as 900 μm and has a two-layer structure with the inner layer attached to epithelial cells but devoid of bacteria and a non-attached loose layer towards the lumen that stains for embedded commensal bacteria (Johansson et al., 2008). That this epithelial surface compartment is a discrete microenvironment and ecological niche is also determined by an oxygen gradient across the epithelium reaching into the lumen. There is a steep oxygen tension gradient within the tissue so that the epithelial cells are already exposed to markedly lower oxygen levels than found in blood. This gradient however reaches across the tissue into the mucus layer and the lumen and could in colon fall from ∼40 mm Hg in the submucosa to 3 mm Hg in the lumen (He et al., 1999). The oxygen present close to the tissue surface may as well explain that anaerobic bacteria (that make up most of the commensal species) will not occupy this compartment. These spatial differences in longitudinal and radial directions concerning bacterial densities but also in the composition of the microbiota need to be taken into account when projecting findings from stool samples into the *in situ* situation in colon. And that applies not only to differences in bacterial signatures but more so when “omics-data” collected from stool samples are interpreted in view of the capacities of the microbiota in the gut. Some care is thus required when interpreting data from a simple stool sample and predicting what it could mean for the host and host health.

## How to better assess the interplay of physiology and microbiome science

Given the limits of rodent studies in understanding the interrelationship of gut responses to environmental cues and microbiome alterations, it may be wise to invest more into well-designed human studies. That should be based on well-characterised individuals with well-characterised microbiota and a well-documented intestinal phenotype. There are hardly any genome-phenome association studies that could help to explain what causes the heterogeneity in gut responses. However, a recent study has identified some genetic variables underlying stool frequency differences in humans (Bonfiglio et al. 2021). The phenotyping should include repeated measures of transit time, average stool volume/weight, wet and dry weight, stool frequency and surrogate markers representing a “healthy gut” such as faecal calprotectin, lactoferrin, S100 protein but also Elastase 1, A1-antitrypsin and faecal fat, eg (Siddiqui et al., 2017). It seems advisable not only to assess relative abundance as done in almost all microbiome studies but also real bacterial counts/densities. That also would provide information on the relationship between stool volume/mass and bacterial biomass contained (vital and dead) which is known to vary across the world but even across Europe (Cummings et al., 1992) and any comparison of microbiomes from rural and industrialised communities has stool mass as a putative confounder. Stool volume/mass passed per day is markedly influenced by the quantity and kind of dietary fibre which secondarily also effects stool water content which in turn associates with microbiome diversity. Studies in healthy volunteers consuming well-defined diets for 20 days with varying fibrecontent and types caused differences in faecal mass of four-fold (from 74 to 288 g/day) and bacterial counts in those samples also increased from 2 × 10^12^ to 9.5 × 10^12^ per day from low to high fibre intake diets possibly by increased substrate availability for growth (Forsum et al., 1990). Future microbiome studies need to collect these quantitative stool data for better understanding of the functional capacities of the microbiota – because in biology “mass matters”. A good basic characterisation of the faecal microbiome requires repeated analysis and those could lead to a sub-clustering by what used to be called “enterotype”. Although the concept of enterotypes has been questioned, a very recent study demonstrated that the outcome of any dietary manoeuvre, eg, depends on the starting point represented by distinct enterotypes (Klimenko et al., 2022).

If possible, healthy volunteers could be paired with ileostomy patients with similar anthropometric features. The ileostomy gives access to the chyme that moves into the large intestine allowing extensive analysis of products of digestion and absorption and of the substrate load entering colon. If the “study twin” is fed the same diet or treated in the same manner, it can be estimated what quantity and quality of residual food constituents normally passes the ileocecal valve. Treatment should always monitor also transit time by use of dyes or other makers. Although the ileostomy increases modestly the bacterial density in ileum over that in normal volunteers (Finegold et al., 1970), its advantage is that basal or meal-stimulated substrate flow can be assessed together with other information such as pre- and postprandial responses in nutrients and hormones. When using the outflow of the stoma as the substrate for add-on *in vitro* fermentation with stool samples collected from the twin, analysis of the products by extended metabolite profiling can be compared to products contained in a stool sample collected. That would allow a better assessment of the input–output relationship – including a proper caloric balance – across the colon and for association with the microbiome-signatures obtained. Such a setting would also allow, eg, the fate of different drugs known to affect the microbiome to be followed and to obtain relevant concentrations and how those in turn alter the microbiome or the metabolome in the gut and stool.

Taken together, analysis of a stool sample with relative abundance data is of limited information quality for the role that the microbiome plays in the large intestine and in the overall health-disease trajectory. Moreover, because we still lack the reference of a “healthy gut microbiome” (Shanahan et al., 2021), options for targeted changes are also limited. Microbiome science often neglects the many physiological variables proven to contribute to the quantity and diversity of the microbiota and that significantly limits proper data interpretation. It seems advisable to collect as much information about gut physiology and functionality such as stool appearance based on BSS, eg, stool frequency, water and dry matter content, bacterial counts in samples – which all have been demonstrated to associate closely with microbiome diversity and the health or disease status.
